# Effect of oral fluids on dental caries detection by the VistaCam

**DOI:** 10.1002/cre2.13

**Published:** 2015-12-29

**Authors:** Fardad Shakibaie, Laurence J Walsh

**Affiliations:** ^1^ School of Dentistry The University of Queensland Brisbane Australia

**Keywords:** dental caries, vistacam, fluorescence, diagnosis

## Abstract

The VistaCam® system (Durr Dental, Bietigheim‐Bissingen, Germany) has been suggested as an adjunct to clinical examination for dental caries. This study assessed whether the digital scores obtained for tooth surfaces were affected by the colour of the carious lesions present and by the presence of saliva or blood on the tooth surface. The VistaCam intra‐oral ries, or with sound enamel and root surfaces, with or without overlying dental plaque biofilm. Teeth that had undergone root treatment or were stained by tetracycline were also assessed. Readings were taken in the dry state and after application of human stimulated saliva or venous blood onto the surface of the samples. VistaCam fluorescence scores for all samples were similar in the dry state, and when covered with saliva (P > 0.05), however a coating of dilute blood elevated the readings for most samples to a high level (P < 0.01), other than tetracycline stained teeth and dental caries (P > 0.05). Readings for healthy enamel were 0.9–1.1, and these increased up to 2.8 in the presence of blood. VistaCam fluorescence scores are not affected by dryness or moisture from the presence of saliva but increase when traces of blood are present. This problem needs to be taken into account when the device is used clinically, because blood from the gingival crevice is a common contaminant of tooth surfaces when patients have widespread gingival inflammation. There are also issues with elevated scores from background fluorescence from tetracyclines, which need to be considered when the system is in clinical use.

Dental caries is one of the most prevalent dental conditions (Petersen et al. 2005), and much emphasis has been placed on the detection of the disease at early stages. Radiographs have been used as an adjunct to clinical examination but suffer from concerns regarding ionizing radiation (White & Pharaoh 2014). There is current interest in optical devices which emit non‐ionizing radiation and are therefore inherently safe, allowing them to be used at frequent intervals to detect and monitor caries. Amongst these innovative technologies are fluorescence detection systems combined with intra‐oral cameras including the VistaCam® (Durr Dental, Bietigheim‐Bissingen, Germany) camera (405 nm wavelength light) and the SoproLife® (Acteon, Merignac, France) (450 nm blue light and visible white light) (Eberhart et al. 2007; Tomczyk et al. 2014).

The 405 nm wavelength violet light emitted by the VistaCam elicits fluorescence from both inorganic and organic components of teeth (Tassery et al. 2013). When imaging in fluorescence mode, structures containing porphyrin molecules, such as caries, stains (Shakibaie & Walsh 2014; Shakibaie & Walsh 2015) and mature dental plaque (Walsh & Shakibaie 2007) emit visible red light when illuminated by violet light (König et al. 1993; Buchalla et al. 2004a; Buchalla et al. 2004b). The violet excitation light is not seen by the camera as it is removed by a filter located in front of the sensor. Image processing (as described in US patents 6,206,691 B1, 7,106,958 B2; and 7,341,450 B2) allows the extent of fluorescence to be mapped using a look‐up table to a pseudocolour image. The colours align with numerical values for the pixels (Jablonski‐Momeni et al. 2013). These data may potentially be used to differentiate lesions of dental caries from tooth structure.

The present study was undertaken to investigate the effects of contaminating saliva and blood on the numerical measurements generated by the system. The null hypothesis was that VistaCam scores would not differ from samples assessed in the dry state and when covered with saliva or blood.

## Materials and Methods

### Experimental design

A total of 112 extracted human teeth from the permanent dentition were collected with the approval of the institutional ethics committee (Reference No: 2003000040) from a dental school exodontia clinic. A power analysis was undertaken to determine sample sizes in the various groups, based on data from a pilot study. The power analysis assumed *α* = 0.05 and estimated *β* = 11.06 (study power = 88.94%). All teeth except those for group A2 were from adults aged 18 years or more and were examined under 20× magnification for pathology or defects. All teeth were carefully cleaned to remove external stains from the enamel. The sample groups comprised the following:

Group A1. Ten premolar teeth from patients aged over 17 years free of plaque, caries and other defects, which had been removed for orthodontic reasons. These were stored in highly diluted sodium hypochorite solution (0.075% available chlorine and 1.24% saline) to prevent any bacterial growth occurring on the samples.

Group A2. Ten premolar teeth from teenage patients aged 17 years or less, free of deposits or disease. These were stored in the same manner as for Group A1.

Group B1. Twenty teeth with large coronal cavities where the carious dentine was darkly coloured (dark brown or black). These were stored in distilled water saturated with thymol.

Group B2. Twenty teeth with large coronal cavities where the carious dentine was not different in colour from sound dentine. These were stored in distilled water saturated with thymol;

Group C1. Twenty teeth with thick deposits of plaque already present on a sound root surface, stored in stimulated human saliva diluted 1:10 in distilled water.

Group C2. Twenty teeth with thick deposits of plaque already present on a sound enamel surface, stored in stimulated human saliva diluted 1:10 in distilled water.

Group D. Five teeth with banded staining of the roots resulting from the use of tetracycline (confirmed by ultraviolet fluorescence examination of the teeth), stored in distilled water with thymol.

Group E. Seven premolar and incisor teeth that had undergone root canal treatment and had been obturated with gutta percha and AH26® epoxy resin sealer (Dentsply, Tulsa, OK, USA), and had darkly stained crowns and roots, and had been stored in distilled water with thymol.

The VistaCam intra‐oral camera was connected via USB to a laptop computer, and the manufacturer's supplied spacer was applied to give consistent position and angulation. The system was used in a dark room to avoid the influence of extraneous light. Each sample was assessed by one examiner in the moist state (without surface water). The image under 405 nm wavelength violet light excitation was recorded and analyzed using the supplied Dürr DBSWIN software, to generate a pseudoreference image, which provided the maximum numerical digital data which were used for subsequent analysis.

To assess the influence of surface factors, normal and pseudoreference images were retaken after covering the samples with 40 μL volumes of human stimulated saliva from a single healthy male volunteer, and then after rinsing this away, with the same volume of heparinized anticoagulated human blood obtained from the same volunteer (diluted 1:8 in distilled water). This was intended to replicate the effect of gingival bleeding, which could occur under clinical conditions, giving contamination of the tooth surfaces by saliva mixed with small amounts of blood.

### Data analysis

The VistaCam numerical scores from the processed pseudoreference images were recorded, and GraphPad Prism (GraphPad Software Inc, La Jolla, CA, USA) version 6 statistical software used to compare sample types, under different conditions (dry, saliva coated and blood coated). Data sets were assessed for normality. A repeated measures one‐way parametric analysis of variance was used to analyze differences between the dry state and the saliva wet state and blood covered state. As the primary purpose of the device is as a caries diagnostic adjunct, light and darkly stained carious dentine groups were then compared to other sample types. The Kruskall–Wallis test was used for comparisons between sample types under the same surface conditions.

## Results

**Figure 1:**
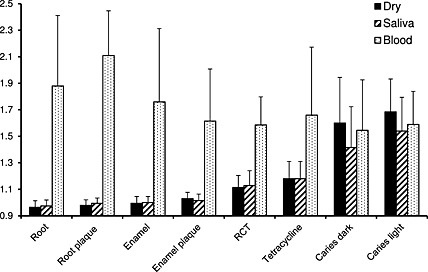
VistaCam data showing means and standard deviations for various tooth samples. The Y axis shows the scores from the system, in arbitrary fluorescence units.

Mean VistaCam scores from samples are shown in Figure 1, while Table 1 presents summary results for numerical ranges and the influence of surface coverings on VistaCam scores.

**Table 1:** VistaCam data for tooth samples under different conditions.

Sample	Dry	Saliva	Blood	Significance
Dark caries	0.9–2.2	0.9–2.1	0.9–2.1	A (NS), B (NS), C (NS)
Light caries	1.2–2.2	0.9–2.1	1.3–2.2	A (NS), B (NS), C (NS)
Sound dentine	0.9–1	0.9–1	0.9–2.7	A (NS), B (*P* <0.0001), C (*P* < 0.0001)
Sound enamel	0.9–1.1	0.9–1.1	1–2.8	A (NS), B (*P* <0.0001), C (*P* < 0.0001)
Plaque on dentine	0.9–1	0.9–1.1	1.5–2.9	A (NS), B (*P* < 0.0001), C (*P* < 0.0001)
Plaque on enamel	1–1.1	0.9–1.1	1–2.6	A (NS), B (*P* < 0.0001), C (*P* < 0.0001)
Tetracycline stained	1.1–1.4	1.1–1.4	1.1–2.3	A (NS), B (NS), C (NS)
RCT discoloured	1–1.2	1–1.3	1.2–1.8	A (NS), B (*P* = 0.0056), C (*P* = 0.004)

Data show the minimum and maximum values for each situation. Differences due to surface conditions (dry vs. saliva vs. blood) for repeated measures analysis of individual samples are designated as follows: A – significant difference for dry versus saliva; B – significant difference for dry versus blood; C – significant difference for saliva versus blood; NS – not significant (*P* > 0.05). According to the manufacturer of the VistaCam, numerical scores above 2.0 correspond to lesions beyond the dentino‐enamel junction, and scores above 2.5 correspond to deep dentine caries. RCT – root canal treatment.

There were no statistically significant differences between dry and saliva‐coated surfaces. Thus, in relation to saliva coating, the null hypothesis was accepted.

**Figure 2:**
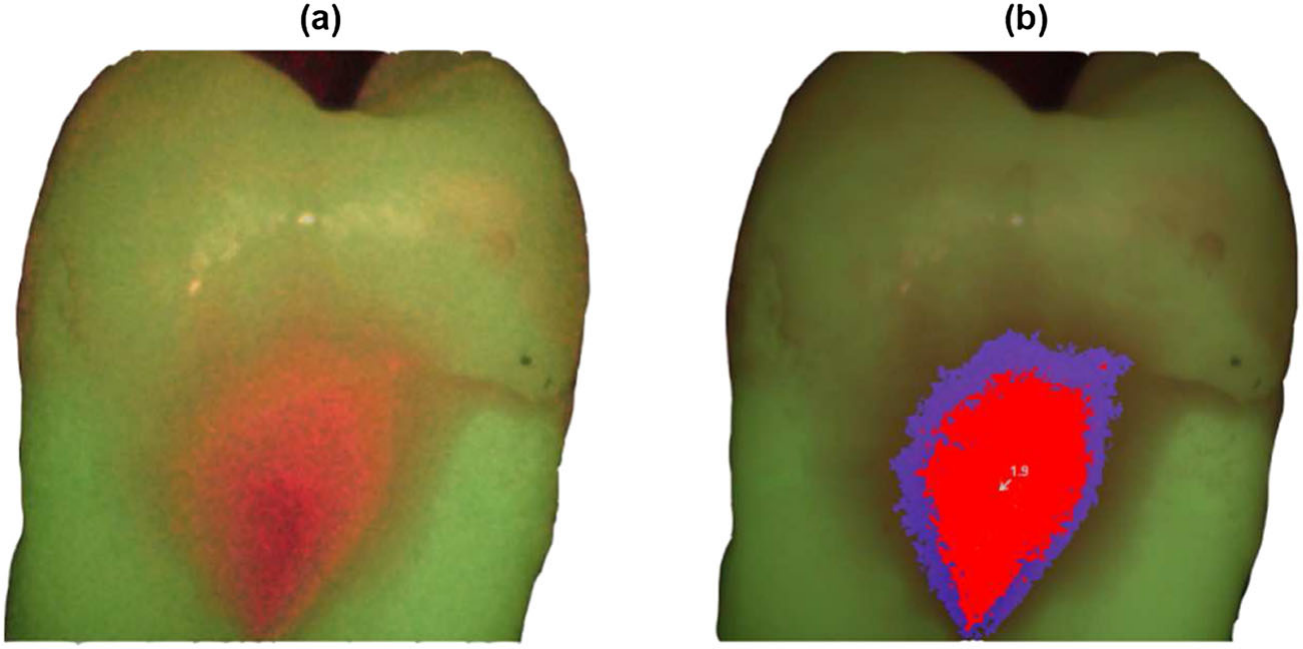
(a) Normal image of a tooth root covered with diluted blood, viewed using the VistaCam. (b) The pseudo reference image of the same image.

In contrast, the presence of blood generated fluorescence, which significantly elevated the originally low scores for healthy and plaque‐covered enamel and dentine, and for discoloured roots, compared to the same materials in a dry state or when coated with saliva, as it has shown this negative red blood imaging for one of the tooth samples covered with plaque in Figure 2. Thus, for these sample types, for blood contamination, the null hypothesis was rejected.

Sample types that already had high scores in the dry state (light caries, dark caries and tetracycline stained teeth) did not show significantly elevated scores when blood was present.

**Figure 3:**
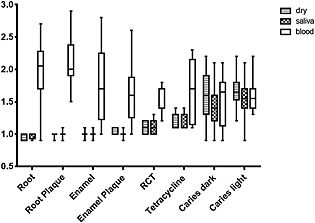
Boxplots showing VistaCam data ranges for various tooth samples.

The effect of sample type on VistaCam scores is summarized in Table 2, and their data distributions are shown in box plots in Figure 3. For samples with dry surfaces, the scores for both dark caries (0.9–2.2) and light caries (1.2–2.2) were similar (*P* > 0.05), and both were significantly higher than those for sound dentine (0.9–1), sound enamel (0.9–1.1), dental plaque on enamel (1–1.1) or roots (0.9–1), tetracycline stained (1.1–1.4) and root treated discoloured teeth (1–1.2). Regions of dental caries gave scores up to 2.2, whilst noncarious samples consistently gave scores in the range of 0.9–1.2 (Table 1). The same trend was seen for surfaces when covered with saliva.

**Table 2:** VistaCam data mean differences for dark and light carious versus other tooth samples.

	Dry		Saliva		Blood	
Sample	Dark Caries	Light caries	Dark caries	Light caries	Dark caries	Light caries
Sound dentine	<0.0001	<0.0001	<0.0001	<0.0001	0.4812	0.5033
Sound enamel	<0.0001	<0.0001	<0.0002	<0.0001	>0.9999	>0.9999
Plaque on dentine	<0.0001	<0.0001	<0.0001	<0.0001	0.0029	0.0031
Plaque on enamel	0.0004	<0.0001	0.0017	<0.0001	>0.9999	>0.9999
Tetracycline stain	>0.9999	>0.9999	>0.9999	>0.9999	>0.9999	>0.9999
RCT discoloured	>0.9999	0.4795	>0.9999	>0.9999	>0.9999	>0.9999

RCT – root canal treatments.

For dry and saliva coated samples, both dark caries and light caries gave significantly elevated scores (*P* < 0.001) over sound roots and roots covered with dental plaque, and the same pattern was seen for sound enamel and enamel with dental plaque. Because of greater inherent sample fluorescence in all samples other than dentine surfaces covered with plaque, the presence of blood elevated the scores, but the gain was not statistically significant (*P* > 0.05).

## Discussion

The manufacturer's instructions for using the VistaCam highlight a number of factors that can affect the fluorescence scores and hence the analysis, namely the remains of food, dental calculus, plaque disclosing dyes, prophylaxis pastes, fluoride pastes and polishing pastes. The influence of saliva or blood on the surface is not however discussed in the instructions for use. In addition, stains present on teeth and restorations would also be expected to influence their fluorescence properties. In the present study, we chose to eliminate the effect of surface stains by removing them from all samples at the start of the study before they were scored using fluorescence, in line with past work (Shakibaie & Walsh 2012) to eliminate influences of external stains on fluorescence readings.

The present results show that VistaCam scores are not affected by the presence of saliva but are elevated by the presence of blood on the surface of the sample. This information provides an important caveat to how the device should be used clinically. The water component of saliva and blood (which in saliva is 99% by volume) (Walsh 2007) does not strongly absorb 405 nm or other visible light wavelengths (Litjens et al. 1999). Clinically, blood from the gingival crevice may be a common contaminant of tooth surfaces when patients have widespread gingival inflammation. The use of other wavelengths of light to elicit fluorescence could possibly avoid such issues caused by blood. Importantly, because visible red light is transmitted through blood, optical methods based on red light could still be used for detecting dental caries in the presence of frank bleeding. This principle has already been demonstrated for dental calculus (Krause et al. 2005).

The digital scores generated by the VistaCam were found to be similar for lesions of dentine caries, regardless of whether they were coloured light or dark. As all the carious lesions in the study had frank surface cavitation, so would have been identified by a competent dental professional during a clinical examination using a mirror and dental probe. Scores for tetracycline teeth were high despite the absence of caries. This stresses the point that high scores for fluorescence on teeth may be caused by factors other than dental caries.

The use of fluorescence‐based methods using violet light combined with a red filter to aiding the identification of infected dentine within cavitated dental caries has been an established method for many years (Hibst & Gall 1998; Buchalla et al. 2004a; Stoll et al. 2015). The technique relies on the endogenous fluorescence properties of bacterial porphyrin derivatives, by‐products and metabolites (Thoms 2006). Because of endogenous fluorescence from tetracyclines, this technique cannot differentiate caries from tetracycline staining, since both fluoresce when excited with violet light. This is a limitation of the violet wavelength chosen and may contribute to less than desirable performance in terms of selectivity and sensitivity when compared to other optical caries detection technologies (Eberhart et al. 2007).

## Conclusion

The fluorescence measurement process used by the VistaCam intraoral camera can distinguish healthy enamel and root surfaces with or without plaque, from those with carious lesions. This discrimination is reliant upon the surface being dry or covered with saliva, because the scores given by the system are elevated considerably by the presence of blood, giving high readings regardless of the nature of the underlying substrate. There are also issues with high readings due to background fluorescence from tetracyclines. These issues need to be considered when the system is in clinical use.

### Clinical relevance

## Scientific rationale for the study

Image processing of images of teeth by the VistaCam can help identify lesions of dental caries in clinical practice. The image processing of fluorescence emissions relies on greater signals being emitted from areas affected by dental caries.

## Principal findings

The presence of small amounts of blood on the surface being examined greatly elevates the fluorescence score. The presence or absence of saliva on tooth surfaces does not alter scores in most situations.

## Practical implications

When using the VistaCam as an adjunct to clinical caries diagnosis, clinicians need to be aware of elevated scores caused by blood on tooth surfaces

## Conflict of interest and source of funding statement

The authors declare that they have no conflict of interests. This study was supported by National Health and Medical Research (NHMRC) and Australian Dental Research Foundation (ADRF). The study was not funded by the manufacturer of the VistaCam system used in the investigation.
